# Evolutionary Divergence of Gene and Protein Expression in the Brains of Humans and Chimpanzees

**DOI:** 10.1093/gbe/evv132

**Published:** 2015-07-10

**Authors:** Amy L. Bauernfeind, Erik J. Soderblom, Meredith E. Turner, M. Arthur Moseley, John J. Ely, Patrick R. Hof, Chet C. Sherwood, Gregory A. Wray, Courtney C. Babbitt

**Affiliations:** ^1^Department of Anatomy and Neurobiology, Washington University Medical School; ^2^Department of Anthropology, Washington University in St. Louis; ^3^Department of Anthropology and Center for the Advanced Study of Human Paleobiology, The George Washington University; ^4^Proteomics and Metabolomics Shared Resource, Duke University School of Medicine; ^5^Center for Genomic and Computational Biology, Duke University; ^6^MAEBIOS-TM, Alamogordo, New Mexico; ^7^Fishberg Department of Neuroscience and Friedman Brain Institute, Icahn School of Medicine at Mount Sinai, New York, New York; ^8^New York Consortium in Evolutionary Primatology, New York, New York; ^9^Department of Biology, Duke University; ^10^Department of Evolutionary Anthropology, Duke University; ^11^Department of Biology, University of Massachusetts Amherst

**Keywords:** RNA-Seq, human brain evolution, chimpanzee, transcriptome, proteome

## Abstract

Although transcriptomic profiling has become the standard approach for exploring molecular differences in the primate brain, very little is known about how the expression levels of gene transcripts relate to downstream protein abundance. Moreover, it is unknown whether the relationship changes depending on the brain region or species under investigation. We performed high-throughput transcriptomic (RNA-Seq) and proteomic (liquid chromatography coupled with tandem mass spectrometry) analyses on two regions of the human and chimpanzee brain: The anterior cingulate cortex and caudate nucleus. In both brain regions, we found a lower correlation between mRNA and protein expression levels in humans and chimpanzees than has been reported for other tissues and cell types, suggesting that the brain may engage extensive tissue-specific regulation affecting protein abundance. In both species, only a few categories of biological function exhibited strong correlations between mRNA and protein expression levels. These categories included oxidative metabolism and protein synthesis and modification, indicating that the expression levels of mRNA transcripts supporting these biological functions are more predictive of protein expression compared with other functional categories. More generally, however, the two measures of molecular expression provided strikingly divergent perspectives into differential expression between human and chimpanzee brains: mRNA comparisons revealed significant differences in neuronal communication, ion transport, and regulatory processes, whereas protein comparisons indicated differences in perception and cognition, metabolic processes, and organization of the cytoskeleton. Our results highlight the importance of examining protein expression in evolutionary analyses and call for a more thorough understanding of tissue-specific protein expression levels.

## Introduction

Despite extensive cognitive specializations and evolutionary changes in brain morphology in humans (Povinelli and Preuss 1995; Sherwood et al. 2008; Fitch et al. 2010), roughly 98.5% of DNA coding regions is identical to our closest living relatives, chimpanzees (Chimpanzee Sequencing and Analysis Consortium 2005; Prüfer et al. 2012). Early evidence demonstrating a high degree of similarity between human and chimpanzee protein sequences led King and Wilson (1975) to suggest that the substantial differences in the behavioral phenotype between these two species are not only the result of changes to the amino acid sequences of proteins but instead may arise from the differential regulation of homologous genes. Therefore, it may be that differential regulation of molecular expression is responsible for the most profound phenotypic divergence between humans and chimpanzees instead of the biochemical changes implicit in sequence evolution. The fact that the human and chimpanzee proteomes differ only by about 50,000 changes in amino acid sequence (Chimpanzee Sequencing and Analysis Consortium 2005) reinforces the plausibility of King and Wilson’s proposal. Indeed, protein–protein interactions, the foundation of cellular molecular function, are potentially affected by changes in amino acid sequence, and therefore alterations to DNA coding regions may have deleterious effects on the biochemical functions of a protein (Goodman 1963; Wray et al. 2003; Fraser et al. 2004). Accordingly, the human behavioral phenotype may have arisen, in part, through changes in the expression levels of gene transcripts and proteins, while keeping the amino acid sequences of proteins relatively stable.

Empirical evidence has revealed profound differences in the regulation of transcriptional expression in the human brain compared with that of the chimpanzee. A survey of promoter sequences found many more *cis*-regulatory sequences were enriched for positive selection in humans compared with chimpanzees and may target the expression of genes supporting neural development and glucose metabolism in particular (Haygood et al. 2007). Additionally, intermolecular gene regulation through *trans*-regulatory elements, specifically microRNAs (miRNAs) or transcription factors, is known to cause divergent patterns of transcript expression between humans and chimpanzees. Novel miRNAs may have emerged within the human lineage as key translational regulators (Berezikov et al. 2006; Hu et al. 2012), and miRNA-mediated gene silencing is enhanced in the human brain compared with other primates (Somel et al. 2011). Furthermore, transcription factor sequences have been shown to evolve more rapidly in humans compared with chimpanzees (Bustamante et al. 2005). When examining the expression of transcripts across brain regions, humans display unique patterns of coexpression compared with chimpanzees, which may underlie species-specific changes in regional connectivity and network dynamics (Oldham et al. 2008; Konopka et al. 2012).

Despite our knowledge of the regulatory mechanisms affecting transcription, it is not well understood how the expression levels of transcripts correspond to downstream protein abundances. The rates of transcription and translation and the differential degradation rates of mRNA and proteins are processes that ultimately affect protein abundance, and each of these steps is governed by strict regulation (Komili and Silver 2008; de Sousa Abreu et al. 2009). Recent studies measuring molecular expression in human or chimpanzee cell lines have found that transcript abundance predicts between 4% and 50% of protein expression (Schwanhäusser et al. 2011; Khan et al. 2013; Wu et al. 2013). However, because these studies were performed in undifferentiated cell culture to control for perturbations that cause measurement error, it is not known whether the relationship between transcript and protein abundance remains similar in differentiated tissue or whether tissue differentiation confounds this relationship further. Moreover, it is unclear to what extent the discordance of expression levels between transcripts and proteins affects the biological signals obtained from enrichment analyses on brain tissue from two closely related species.

In this study, we explore the relationship between the expression of gene transcripts to proteins of humans and chimpanzees in two regions of the brain, the anterior cingulate cortex (ACC) and the caudate nucleus (CN). We used RNA-Seq and ultraperformance liquid chromatography coupled with high-resolution accurate mass tandem mass spectrometry (LC/MS/MS) on the same samples of brain tissue for the identification and quantification of transcripts and proteins, respectively. The ACC is a region of the neocortex that is among the most enlarged in human evolution (Hill et al. 2010; Fjell et al. 2013). Activity in the ACC is involved in cognitive processes, including executive control (Kerns et al. 2004), attention (Pardo et al. 1990), and visual perception of spatial relationships among objects (Fjell et al. 2013). The CN is a subcortical structure of the basal ganglia, which contains a large population of medium spiny neurons that primarily release the inhibitory neurotransmitter GABA, unlike the predominantly glutamatergic neurons of the cerebral cortex (Tepper et al. 2010). The CN is implicated in the execution of movement, goal-directed action, memory, learning, and the production of speech in humans (Jarvis 2004; Pfenning et al. 2014). These regions of interest were selected for this study as they are expected to have a large degree of divergence in molecular expression between the two species due to their roles in human-specific cognition, but the molecular expression profiles between the two regions may differ considerably as components of the neocortex and basal ganglia.

Our study had two main objectives. First, we tested the hypothesis that the relationship between the expression of transcripts and proteins differs by species and region of the brain. We found lower correlations in the abundances of gene transcripts to proteins than previous studies utilizing undifferentiated cell lines, suggesting that the relationship in the expression of these molecules is particularly divergent in brain tissue of humans and chimpanzees. Second, we examined whether differential enrichment analyses of transcripts and proteins revealed the same biological signals between the two species. To address these issues, we performed differential expression analyses on the complete transcriptional and proteomic data sets, but we also constructed a data set where transcripts were paired with their protein products, creating a 1:1 ratio of transcripts and proteins. This strategy enabled us to determine whether differences in biological signals were the result of the greater molecular coverage of transcriptional analyses. Although some categories of biological function were differentially expressed (DE) in both types of molecules, we found that there were certain signals to which transcripts or proteins are uniquely sensitive. This study further supports the perspective that transcript and protein expression data are not interchangeable (Warnefors and Kaessmann 2013) and that the biological signals accessible by each molecule should be considered when designing studies of comparative molecular expression.

## Materials and Methods

### Samples

Frozen human brain samples (aged 34–51 years) were obtained from the National Institute of Child Health and Human Development Brain and Tissue Bank for Developmental Disorders at the University of Maryland (Baltimore, MD) and were free from neurological disorders. Frozen brain samples from adult common chimpanzees, *Pan troglodytes* (aged 23 to 35 years), were obtained from the Alamogordo Primate Facility (Holloman Airforce Base, Alamogordo, NM). The chimpanzees had been cared for according to Federal and Institutional Animal Care and Use guidelines and died of natural causes. ACC and CN were sampled from three adult humans and three adult chimpanzees. ACC samples were dissected near the genu of the corpus callosum, corresponding to Brodmann’s area 24, and contained all neocortical layers and a small amount (<10%) of underlying white matter. CN samples were dissected from the head of the caudate and contained no surrounding white matter. All samples were divided into two pieces for RNA sequencing and for quantitative proteomics, respectively. The tissue was collected and stored at −80 °C with postmortem intervals of less than 8 h to diminish degradation of proteins. A detailed summary of the sample, including ages and sexes of individuals, is provided in supplementary table S1, Supplementary Material online.

### Transcriptome and Proteome Generation

Total RNA was isolated with an RNeasy kit (Qiagen, Valencia, CA) including a DNaseI treatment step. Four micrograms of total RNA was used to make each transcriptome library. Library construction was performed with the Illumina Tru-Seq kit (Illumina, San Diego, CA). Libraries were sequenced at the Institute for Genome Sciences & Policy and the Genome Sequencing & Analysis Core Facility at Duke University. Approximately 30 million 50-bp sequences were produced for each library. Orthologous gene models for each species were constructed using methods described previously (Blekhman et al. 2010). Sequences were mapped to the species-specific genomes, human (hg19) and chimpanzee (panTro3) (Trapnell et al. 2009). Gene transcripts were quantified in counts per million using HT-Seq (http://www.huber.embl.de/users/anders/HTSeq/doc/overview.html, last accessed July 22, 2015), and the data were normalized using edgeR (Robinson et al. 2010).

The Proteomics Core Facility at Duke University prepared and performed LC/MS/MS on all the samples for protein identification and quantification. Details regarding these procedures and their reproducibility can be found in the supplementary text and figures S1 and S2, Supplementary Material online. Proteins were quantified in summed ion intensity, and the resulting proteomic data set was normalized using the same method as the genomic data.

### Data Set Construction

The Synergizer (http://llama.mshri.on.ca/synergizer/translate/, last accessed July 22, 2015) was used to match proteins back to their parent transcripts by searching the Ensembl database. This produced a list of 791 transcript–protein pairs. Several genes matched to more than one protein product, typically different isoforms of the same protein. In this case, the protein with the highest Teller score (confidence rating of the protein assignment) and an assigned function in the UniProt database (http://www.uniprot.org, last accessed July 22, 2015) was kept in the data set. For inclusion in this study, each human protein had to have a chimpanzee homolog (Uniprot identification ending with “PANTR”). The resulting list contained 715 homologous proteins, each paired to their theoretical transcript parent. For simplicity, we referred to proteins by their human identifier (Uniprot identification ending with “HUMAN”).

### Variation in Gene and Protein Expression

We explored intraspecific variation of gene and protein expression by finding the coefficient of variation (CV) across the three individuals per species. Because CVs have no units and are normalized to the mean of the species-specific expression level, interindividual variance can be compared between the two sets of molecular data. Mann–Whitney tests were performed to examine whether the central tendencies of the interindividual CVs of the molecules differed, and Kolmogorov–Smirnov tests were used to determine whether their distributions differed in terms of shape.

### Differential Expression

To explore possible functional implications of transcript and protein expression, we performed categorical enrichment analyses on Gene Ontology (GO) categories of biological function (Gene Ontology Consortium 2000) using pyEnrichment (https://github.com/ofedrigo/pyEnrichment, last accessed July 22, 2015). The background was all of the transcripts or proteins of the data set. Significance levels for difference in expression were determined with a modified exact test similar to Fisher’s exact test.

### Regression Analyses

The relationship between the expression levels of parent transcripts and their protein products was explored using species means. We performed ordinary least squares (OLS) regressions, which account for error present in the *y* dimension (Smith 2009). We opted against performing reduced major axis (RMA) regressions, which purports to diminish the variance along the *x*- and *y* axes (Sokal and Rohlf 1995). Some authors have suggested that the error accounted for by RMA along the *x* axis can originate from biological sources in addition to error implicit in measurement (Kelly and Price 2004; Hansen and Bartoszek 2012). All regressions were calculated using SMATR package (version 3.3) for R (version 3.0.1) (Falster DS, Warton DI, and Wright IJ, https://github.com/dfalster/smatr/, last accessed July 22, 2015). To explore whether transcript and proteins pairs supporting disparate biological functions differ in their scaling relationships, we ran OLS regressions on the average species expression of transcripts and proteins supporting GO categories of biological function (484 categories in ACC, 485 in CN).

## Results

### Genomic and Proteomic Data Sets

In total, we assayed expression from 12,443 gene transcripts in the ACC and 11,787 genes in the CN of humans and chimpanzees. The proteomic data set was based on the expression of 8,775 peptides from 1,337 proteins. The quantitative data for each sample at the peptide and protein-level can be found in supplementary data set S1, Supplementary Material online. This file also contains individual expression levels for each transcript and protein, the species mean, standard deviation, and interindividual CV.

Because our goal was to assess the biological signals from transcripts that could be compared directly with their corresponding proteins, and vice versa, we constructed a “paired” data set, consisting of theoretical transcript parents each paired to a single protein product (522 pairs in the ACC, 499 in the CN once one outlier was removed from the analysis in each region [see below]). However, we performed the same analyses on the “unpaired data sets,” which consisted of the entire sets of transcripts and proteins that were quantified and had homologs in the chimpanzee. Results and discussion of the unpaired data set can be found in the supplementary text, Supplementary Material online. Table 1 lists the numbers of transcripts and proteins in each of these data sets.

**Table 1:** The Number of Transcripts and Proteins that Are Uniquely Identified or Those that Can Be Paired As a Single Gene Transcript with a Protein Product

	ACC	CN
Total transcripts	12,443	11,787
Uniquely identified transcripts, “unpaired”	11,920	11,287
Total proteins	715	715
Uniquely identified proteins, “unpaired”	192	215
“Paired” transcripts and proteins	523	500

As expected, many transcripts did not have a corresponding protein that could be measured (11,920 in ACC and 11,287 in CN). However, a surprisingly large number of proteins were analyzed that did not have matching RNA transcripts detected (192 in ACC and 215 in CN). The gene models for each of these transcripts were included in the list of orthologous protein-coding regions that we attempted to detect using RNA-Seq (see Materials and Methods). In order to further explore this discrepancy between data sets, we performed an enrichment analysis on these proteins using DAVID Bioinformatics Resource (version 6.7; http://david.abcc.ncifcrf.gov, last accessed July 22, 2015). We found that many of the proteins that did not have a corresponding transcript analyzed were involved in mitochondrial function and metabolism (supplementary table S2, Supplementary Material online). Although this result is somewhat surprising because both transcripts and proteins supporting metabolism are known to be rather stable molecules within mammalian cells (Schwanhäusser et al. 2011), we suspect that rapid postmortem degradation of mRNAs associated with metabolic functions may have caused this effect (Gallego Romero et al. 2014).

### Variation in Transcript and Protein Expression

**Fig. 1.—:**
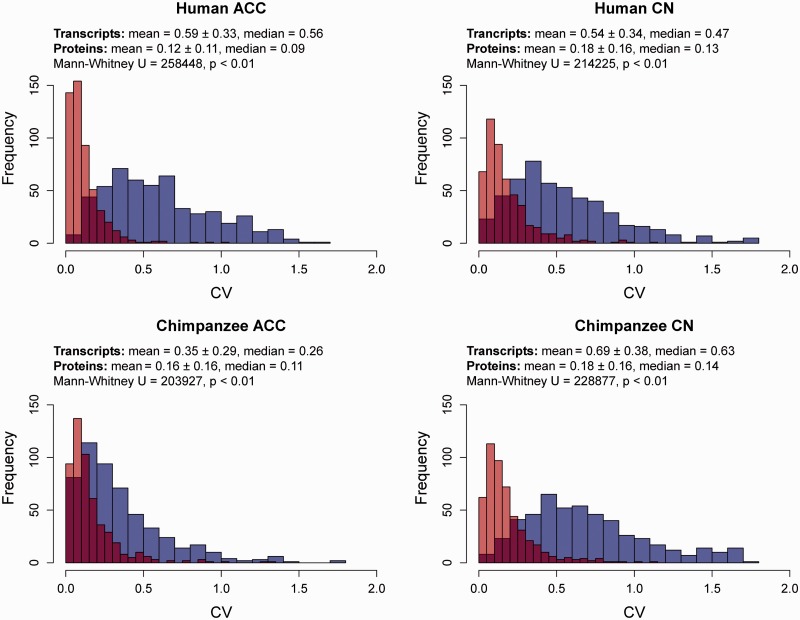
The frequency bar graphs of interindividual CVs for transcript (blue) and protein (red) expression in ACC and CN in humans and chimpanzees using the paired data sets. The overlap between these two distributions appears as a darker (purplish) color. The results of Mann–Whitney tests comparing the central tendencies of transcript and protein expression are provided.

We examined interindividual CVs in transcript and protein expression to determine how the variation in expression levels may differ between transcripts and proteins. The frequency distributions of the CVs for the paired data set of each of these regions are shown in figure 1 and summarized in table 2. In each case, the variation in the expression levels of the transcripts was significantly greater than that of the proteins, and the shape of the distribution of CVs between genomic and proteomic data differed significantly. These data indicate that the expression of proteins is less variable and more constrained than the expression of transcripts in both species and in both brain regions, a result that is consistent with research from primate cell lines (Khan et al. 2013).

**Table 2:** The Results of Mann–Whitney and Kolmogorov–Smirnov Tests of Interindividual CVs between Gene and Protein Expression, Regions of the Brain, and Species in the Paired Data Set

		Mann–Whitney		Kolmogorov–Smirnov	
Comparison		*U*	*P* Value	*D*	*P* Value
Genes versus proteins	Human ACC	2.6 x 10^5^	<0.001	0.76	<0.001
	Human CN	2.1 x 10^5^	<0.001	0.60	<0.001
	Chimpanzee ACC	2.0 x 10^5^	<0.001	0.39	<0.001
	Chimpanzee CN	2.3 x 10^5^	<0.001	0.71	<0.001
ACC versus CN	Human genes	1.2 x 10^5^	<0.01	0.09	0.03
	Chimpanzee genes	2.0 x 10^5^	<0.001	0.46	<0.001
	Human proteins	1.7 x 10^5^	<0.001	0.21	<0.001
	Chimpanzee proteins	1.5 x 10^5^	<0.001	0.12	0.001
Humans versus chimpanzees	Genes in ACC	2.0 x 10^5^	<0.001	0.36	<0.001
	Genes in CN	9.5 x 10^4^	<0.001	0.19	<0.001
	Proteins in ACC	1.1 x 10^5^	<0.001	0.14	<0.001
	Proteins in CN	1.2 x 10^5^	0.70	0.04	0.90

We explored whether there were differences in the variance of molecular expression between ACC and CN. A greater median variance in transcript expression was found in human ACC compared with human CN, whereas chimpanzee CN displayed a greater median variance than chimpanzee ACC. Other studies have found the transcriptional expression of the basal ganglia to be less variable compared to the neocortex of humans and chimpanzees (Khaitovich et al. 2004; Hawrylycz et al. 2012), so it is surprising to find as much variation in the expression of transcripts within the chimpanzee’s CN. Interregional protein expression in humans and chimpanzees displayed a greater median variance in CN in both species. Furthermore, the shape of the distribution of interregional variance in gene transcript expression and protein expression was the same in the ACC and CN. A relatively high level of variation in protein expression in the CN compared with the ACC in both species was unexpected. Although gene transcript expression is relatively stable in the basal ganglia compared with the neocortex (Khaitovich et al. 2004), our results suggest that the opposite may be true for proteins.

There was no clear pattern in interindividual expression between humans and chimpanzees in either molecule or region of the brain. The comparison of variation in the expression of transcripts between species revealed that humans exhibit a greater median variance in ACC, but chimpanzees display a greater median variance in the CN. The shapes of these distributions in variance were also different. Although chimpanzees displayed a greater median variance in protein expression in ACC, the variances in protein expression within the CN were indistinguishable between the two species. Similarly, although the shape of the distribution in variance of protein expression differed between human and chimpanzee ACC, CN exhibited a similar shape of distribution of variance between the two species. These results indicate that the variation in molecular expression is not systematically greater in either species regardless of whether transcripts or proteins are considered. However, the ACC and CN produced different results in these analyses, suggesting that the expression of both transcripts and proteins is influenced by region-specific mechanisms that may result in specialized cognitive functions of the ACC and CN.

### Differential Expression of Genes and Proteins

We compared the mean expression levels of transcripts and proteins in ACC and CN for each species separately by OLS regression analysis using log-transformed data. We discovered one outlier transcript in each comparison: *FHDC1* in human ACC and *AUH* in chimpanzee CN. Although their expression levels were within the range of other proteins (for ACC and CN in both species, Shapiro–Wilk test *P* value < 0.0001; human ACC variability = 5.00 ± 0.62; human CN variability = 5.01 ± 0.61; chimpanzee ACC variability = 4.97 ± 0.64; chimpanzee CN variability = 5.01 ± 0.62), both displayed much lower transcript expression levels than the rest of the transcript–protein pairs (*FHDC1* in the human ACC = median − lower quartile [Q1] * 11.57; *AUH* in the chimpanzee CN = median − Q1 * 13.07). Because we could not ascertain that their low transcript expression levels were due to biological variation and not to measurement error, *AUH* and *FHDC1* were excluded from further analyses.

**Fig. 2.—:**
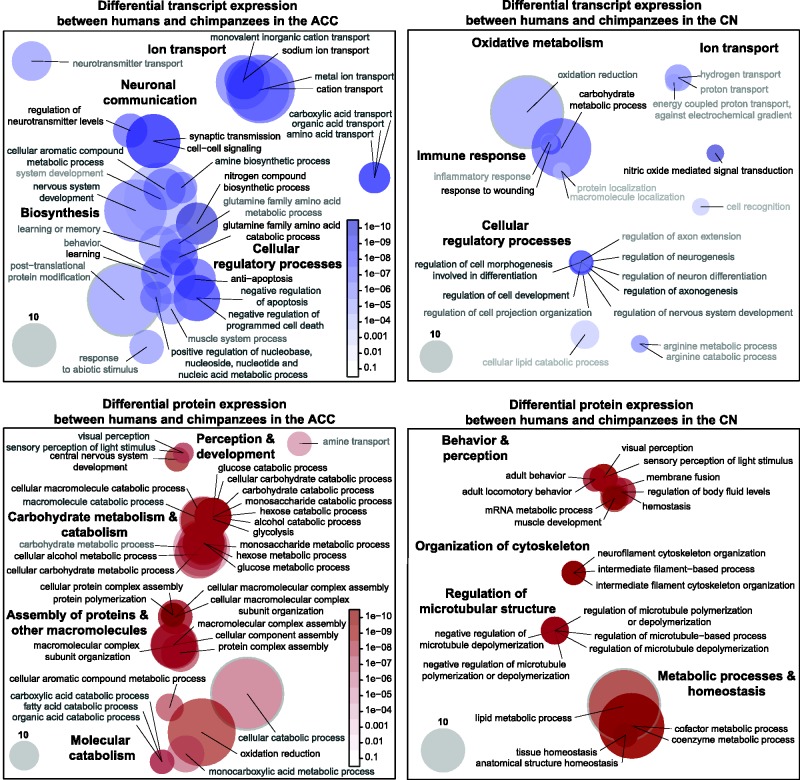
DE gene transcripts and protein products between humans and chimpanzees in GO categories of biological function for the paired data set. The DE categories of transcripts (upper row) are depicted by blue circles for the ACC (upper left) and CN (upper right). The DE categories of proteins (lower row) are depicted by red circles for the ACC (lower left) and CN (lower right). The circles represent categories of biological function, which contain gene transcripts that are DE between the two species. The size of the circle represents the number of genes with a *q* value below the maximum threshold (the gray circles in the bottom left corners provide a guide). The darkness of the circle represents the level of significance (as indicated by the scales, which are the same for both ACC and CN). Aside from the degree of overlap of functional categories, the arrangement of the circles has no meaning. The minimum thresholds are different for genes (in ACC, minimum of five genes per category, *q* ≤ 0.05; in CN, minimum of three genes per category, *q* ≤ 0.05) and proteins (in ACC, minimum of five proteins per category, *q* ≤ 0.05; in CN minimum of three proteins per category, *q* ≤ 0.05).

From the paired data set, we found 36 of 523 transcripts to be DE between humans and chimpanzees in the ACC (FDR ≤ 0.05) and 42 of the 523 proteins to be DE in the same region. In the CN, 33 of the 500 transcripts were DE (FDR ≤ 0.05), and 37 proteins were DE. We performed enrichment analyses on the paired data set to determine whether the expression of transcripts reflected the same biological signals as their protein products. Transcripts supporting 51 categories of biological function in ACC and 22 in CN were DE between humans and chimpanzees (minimum of three genes per category, *q* ≤ 0.05). Biological functions that were DE among the transcripts in the ACC could be broadly categorized as supported neuronal communication, ion transport, cellular regulatory processes, and biosynthesis (fig. 2). In the CN, biological functions that were DE included those involved in oxidative metabolism, ion transport, cellular regulatory processes, and immune response. The list of the significant results of differential expression analyses of the paired data set can be found in table 3.

**Table 3:** DE Gene Transcripts and Protein Products between Humans and Chimpanzees in the GO Category of Biological Function in ACC and CN for the Paired Data Set (Minimum Three Molecules per Category, *q* ≤ 0.05)

GO Biological Process Category	*q* Value	Total Occurrences		GO Biological Process Category	*q* Value	Total Occurrences
**DE transcripts between humans and chimpanzees in the ACC**				**DE proteins between humans and chimpanzees in the ACC**		

Nitrogen compound biosynthetic process	2.10E-03	9		Central nervous system development	4.18E-03	7
Nitric oxide mediated signal transduction	5.25E-03	3		Cellular protein complex assembly	4.33E-03	7
Antiapoptosis	7.67E-03	9		Protein polymerization	7.13E-03	6
Cell–cell signaling	8.41E-03	15		Cellular macromolecule catabolic process	8.93E-03	25
Synaptic transmission	8.41E-03	15		Hemostasis	9.40E-03	4
Sodium ion transport	8.44E-03	8		Regulation of body fluid levels	9.40E-03	4
Glutamine family amino acid catabolic process	9.55E-03	7		Carbohydrate catabolic process	1.01E-02	16
Regulation of neurotransmitter levels	1.04E-02	6		Cellular carbohydrate catabolic process	1.01E-02	16
Cation transport	1.11E-02	24		Oxidation reduction	1.09E-02	54
Learning	1.26E-02	5		Protein complex assembly	1.26E-02	17
Neurotransmitter biosynthetic process	1.35E-02	3		Cellular macromolecular complex assembly	1.48E-02	14
Metal ion transport	1.35E-02	19		Cellular macromolecular complex subunit organization	1.48E-02	14
Response to light stimulus	1.49E-02	3		Cellular component assembly	1.48E-02	25
Nervous system development	1.51E-02	20		Alcohol catabolic process	1.52E-02	15
Hemostasis	1.55E-02	4		Glucose catabolic process	1.52E-02	15
Regulation of body fluid levels	1.55E-02	4		Hexose catabolic process	1.52E-02	15
Amine biosynthetic process	1.71E-02	5		Monosaccharide catabolic process	1.52E-02	15
Catecholamine metabolic process	1.72E-02	3		Macromolecular complex subunit organization	1.54E-02	26
Dopamine metabolic process	1.72E-02	3		Macromolecular complex assembly	1.64E-02	24
Phenol metabolic process	1.72E-02	3		Monosaccharide metabolic process	1.64E-02	21
Monovalent inorganic cation transport	1.76E-02	15		Cellular carbohydrate metabolic process	1.69E-02	28
Amino acid transport	1.79E-02	5		Cellular aromatic compound metabolic process	1.73E-02	9
Carboxylic acid transport	1.79E-02	5		Glycolysis	1.76E-02	13
Organic acid transport	1.79E-02	5		Cellular alcohol metabolic process	1.78E-02	27
Regulation of neurological system process	1.85E-02	4		Glucose metabolic process	1.83E-02	20
Regulation of synaptic transmission	1.85E-02	4		Hexose metabolic process	1.83E-02	20
Regulation of transmission of nerve impulse	1.85E-02	4		Nuclear transport	1.86E-02	3
Positive regulation of nucleobase, nucleoside, nucleotide, and nucleic acid metabolic process	2.04E-02	5		Nucleocytoplasmic transport	1.86E-02	3
Negative regulation of apoptosis	2.12E-02	11		Protein homooligomerization	2.20E-02	4
Negative regulation of programmed cell death	2.12E-02	11		Protein oligomerization	2.20E-02	4
Cellular aromatic compound metabolic process	2.33E-02	9		Monocarboxylic acid metabolic process	2.37E-02	16
Muscle contraction	2.47E-02	4		Aromatic compound catabolic process	2.62E-02	3
Posttranslational protein modification	2.57E-02	27		Cellular catabolic process	2.63E-02	62
Neurotransmitter transport	2.78E-02	8		Sensory perception of light stimulus	2.74E-02	5
Nitric oxide biosynthetic process	2.85E-02	4		Visual perception	2.74E-02	5
**DE transcripts between humans and chimpanzees in the ACC**				**DE proteins between humans and chimpanzees in the ACC**		

Nitric oxide metabolic process	2.85E-02	4		Neurotransmitter metabolic process	3.09E-02	4
Memory	2.97E-02	3		Cellular response to stress	3.36E-02	3
Cell-substrate adhesion	3.38E-02	4		Carboxylic acid catabolic process	3.54E-02	7
Negative regulation of RNA metabolic process	3.39E-02	3		Fatty acid catabolic process	3.54E-02	7
Negative regulation of transcription, DNA-dependent	3.39E-02	3		Organic acid catabolic process	3.54E-02	7
Neurofilament cytoskeleton organization	3.42E-02	3		Macromolecule catabolic process	3.74E-02	27
Glutamine family amino acid metabolic process	3.45E-02	8		Carbohydrate metabolic process	3.77E-02	38
Positive regulation of immune system process	3.49E-02	3		Amine transport	4.32E-02	7
Behavior	4.13E-02	15		Response to inorganic substance	4.45E-02	3
Muscle system process	4.16E-02	5		Response to metal ion	4.45E-02	3
Learning or memory	4.17E-02	9		Muscle development	4.65E-02	3
Cell-matrix adhesion	4.30E-02	3				
Response to abiotic stimulus	4.30E-02	6				
Regulation of neuronal synaptic plasticity	4.35E-02	3				
Regulation of synaptic plasticity	4.35E-02	3				
System development	4.81E-02	27				

**DE transcripts between humans and chimpanzees in the CN**				**DE proteins between humans and chimpanzees in the CN**		

Nitric oxide mediated signal transduction	3.48E-04	3		Muscle development	1.41E-02	3
Carbohydrate metabolic process	1.79E-03	35		Lipid metabolic process	1.57E-02	27
Response to wounding	2.62E-03	4		MRNA metabolic process	1.86E-02	4
Regulation of axonogenesis	4.05E-03	5		Coenzyme metabolic process	2.36E-02	22
Regulation of cell development	4.05E-03	5		Cofactor metabolic process	2.36E-02	22
Regulation of cell morphogenesis involved in differentiation	4.05E-03	5		Sensory perception of light stimulus	2.39E-02	4
Oxidation reduction	1.16E-02	52		Visual perception	2.39E-02	4
Regulation of cell projection organization	1.41E-02	6		Anatomical structure homeostasis	2.82E-02	3
Regulation of nervous system development	1.41E-02	6		Tissue homeostasis	2.82E-02	3
Regulation of neurogenesis	1.41E-02	6		Intermediate filament cytoskeleton organization	2.92E-02	3
Regulation of neuron differentiation	1.41E-02	6		Intermediate filament-based process	2.92E-02	3
Arginine catabolic process	1.91E-02	3		Neurofilament cytoskeleton organization	2.92E-02	3
Arginine metabolic process	1.91E-02	3		Negative regulation of microtubule depolymerization	3.83E-02	4
Regulation of axon extension	2.10E-02	4		Negative regulation of microtubule polymerization or depolymerization	3.83E-02	4
Hydrogen transport	2.48E-02	7		Regulation of microtubule depolymerization	3.83E-02	4
Proton transport	2.48E-02	7		Regulation of microtubule polymerization or depolymerization	3.83E-02	4
Inflammatory response	2.88E-02	3		Regulation of microtubule-based process	3.83E-02	4
Energy coupled proton transport, against electrochemical gradient	4.00E-02	3		Adult behavior	4.14E-02	3
Cell recognition	4.65E-02	3		Adult locomotory behavior	4.14E-02	3
Cellular lipid catabolic process	4.74E-02	8		Membrane fusion	4.54E-02	4
Macromolecule localization	4.88E-02	3		Hemostasis	4.80E-02	4
Protein localization	4.88E-02	3		Regulation of body fluid levels	4.80E-02	4

A similar number of biological functions met our threshold criteria for differential expression between humans and chimpanzees when analyzing proteins as compared with transcripts (46 in ACC and 22 in CN; minimum of 3 proteins per category, *q* ≤ 0.05). Using the listed thresholds, we found that a lower percentage of transcripts and proteins were DE between humans and chimpanzees in CN compared with ACC (transcripts: 10.3% in ACC, 4.4% in CN; proteins: 8.8% in ACC, 4.4% in CN). Importantly, biological functions that were DE in the proteins between humans and chimpanzees in the ACC included those supporting oxidative metabolism, anaerobic metabolism and biosynthesis, perception, and immune response (fig. 2). In the CN, DE biological functions supported biosynthesis, ion homeostasis, perception, and immune response.

Comparing the biological signals accessible by transcripts and proteins within the same brain regions of interest reveals that transcripts are uniquely indicative of cellular regulatory processes, neuronal communication, and immune response, whereas proteins exhibit differences related to organization of the cytoskeleton and molecular catabolism. Our results indicate variability in the brain tissue-specific biological processes that are assessed by either transcriptomic or proteomic analyses, and these findings are largely consistent with the more general functional characteristics attributed to transcripts and proteins with regard to their molecular stability in cell lines (Schwanhäusser et al. 2011).

### Covariance of Gene and Protein Expression

OLS regressions revealed weak, but significant, relationships between gene expression and protein expression in human ACC (β = 0.16, *y*-intercept = 4.64, *R*^2 ^= 0.03, *P* < 0.01, slope confidence interval [CI] = 0.08–0.23), human CN (β = 0.15, *y*-intercept = 4.69, *R*^2 ^= 0.03, *P* < 0.01, slope CI = 0.07–0.21), chimpanzee ACC (β = 0.17, *y*-intercept = 4.58, *R*^2 ^= 0.04, *P* < 0.01, slope CI = 0.10–0.23), and chimpanzee CN (β = 0.14, *y*-intercept = 4.70, *R*^2 ^= 0.03, *P* < 0.01, slope CI = 0.06–0.21). These four regression slopes are significantly less than 1 (*P* < 0.01), indicating a lack of equivalency between the expression levels of genes and proteins. These slopes were subsequently used in comparisons with the scaling of categories of GO biological function (see below) and will hereafter be referred to as “baseline slopes.”

**Fig. 3.—:**
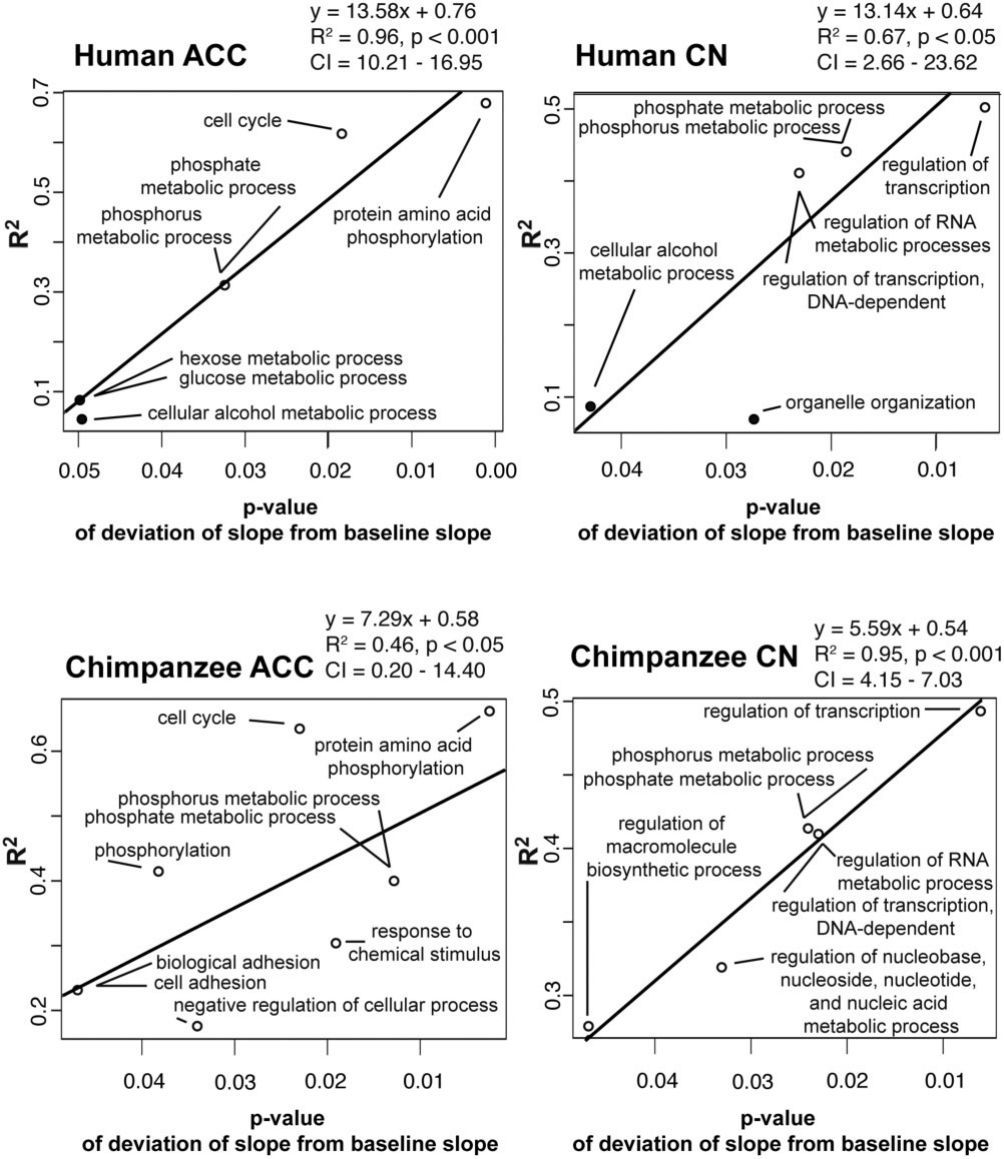
Linear regressions of the *R*^2^ and *P* values of the GO categories of biological function that were significantly different from their baseline slopes. GO biological categories (*n* ≥ 10 gene transcript–protein product pairs) that were significantly different (*P* ≤ 0.05) from the local transcript and protein expression baseline slopes (human ACC β = 0.16, CN β = 0.15; chimpanzee ACC β = 0.17, CN β = 0.14) are plotted with their *R*^2^ values against their *P* value for both regions of interest in humans and chimpanzees. White circles mean that the biological category had a greater slope than the baseline slope, whereas black circles represent a negative slope. The relationship among the points is found by OLS.

To explore whether there is variability in the relationships of transcripts and proteins that support disparate biological functions, we performed separate OLS linear regressions on the expression of transcript–protein pairs from categories of biological function with ten or more transcript/protein pairs per category (484 categories in ACC, 485 in CN; supplementary table S3, Supplementary Material online). Although most categories had a slope similar to that of baseline slopes, the range of slopes was highly variable (human ACC interquartile range [IQR] = 0.05–0.33, range = −0.37 to 0.86; human CN IQR = 0.00–0.28, range = −0.42 to 1.06; chimpanzee ACC IQR = 0.11–0.36, range = −0.62 to 1.20; chimpanzee CN IQR = 0.00–0.27, range = −0.46 to 1.00). These results reveal that different relationships exist between transcript and protein expression depending on biological function. Moreover, a pattern emerged in the data, which to our knowledge has not been reported elsewhere. For biological function categories that deviated significantly from the region- and species-specific baseline slope (*P* ≤ 0.05), we plotted the correlation (R^2^) between gene and protein expression against the *P* value of the categorical slope’s deviation from the baseline. The more the slope of a category of biological function deviated from the baseline, the higher the correlation between gene and protein expression (fig. 3). In each region and species, biological functions that support this observation are involved in transcription, protein modification, and metabolic processes. It is noteworthy that each of these biological processes affects protein abundance (or, in the case of protein modification, how detectable the proteins are to analysis).

## Discussion

We found an overall weak but significant relationship between the expression levels of gene transcripts and protein products in ACC and CN in humans and chimpanzees. These results support other studies in which gene expression levels proved to be poor predictors of protein expression levels in human (Ramakrishnan et al. 2009; Khan et al. 2013; Wu et al. 2013), and chimpanzee and macaque cell lines (Khan et al. 2013). To some extent, a direct correspondence between transcript and protein expression is not expected because the efficiency of translation and rate of protein degradation affect protein availability, causing protein expression levels to deviate from what would be predicted based on transcript abundance (de Sousa Abreu et al. 2009). Moreover, it was recently reported that the effect of some regulatory genetic variants may be buffered at the protein level, despite showing robust effects at the level of the transcript (Battle et al. 2015). We found that there was not a systematic manner by which expression levels of transcripts related to their protein products. The lack of a predictive relationship between transcript and protein expression is a trait shared by both humans and chimpanzees in both ACC and CN. Additionally, our finding of lower interindividual variation in protein expression compared with transcript expression in the brain implies that translation is under stronger stabilizing selection than transcription in both of these species (Schwanhäusser et al. 2011; Khan et al. 2013). However, despite the smaller amount of variation among proteins across individuals, a proportionally greater number of proteins are DE between humans and chimpanzees. This finding implies that even very small differences in protein abundance may be associated with substantial phenotypic divergence.

Our analysis of differential gene and protein expression in the human and chimpanzee brain showed that quite divergent results are obtained when considering the abundances of gene transcripts compared with proteins. Although transcripts are uniquely reflective of cellular regulatory processes, neuronal communication, and immune response, proteomic analyses are better able to detect differences in organization of the cytoskeleton and molecular catabolism. The fact that proteins related to cell structure are DE between species is not surprising because high-throughput proteomic methods tend to measure the most abundant proteins, omitting those that are less prevalent, so to some extent this result is a function of a limitation in proteomic techniques. However, these results are important as they emphasize that different biological signatures are accessible between humans and chimpanzees depending on what type of molecule is examined. It is also worth considering that transcripts degrade at variable rates that can be tied to biological function, with transcripts supporting immune function, for example, degrading very quickly (Gallego Romero et al. 2014). However, whether proteins supporting different functional processes degrade at different rates in postmortem tissue remains unknown.

We found divergent molecular signatures in DE between ACC and CN. Although neuronal communication, biosynthesis, and carbohydrate metabolism are DE in molecular expression from the ACC, oxidative metabolism, immune response and perception are more divergent in CN. In an analysis of gene transcript coexpression networks in humans and chimpanzees, ACC and CN were found to share a similar pattern of expression, potentially indicative of the neural connections between these two regions (Oldham et al. 2006). Although our investigation does not include an outgroup by which to interpret the direction of selection, the biological implications of DE of transcripts and proteins supporting these biological functions should be considered. Specifically, DE of molecules supporting neuronal communication and carbohydrate metabolism in the ACC may underlie alterations in synaptic transmission and energy needs between human and chimpanzee ACC function (Uddin et al. 2004). Notably, these DE categories of biological function are similar to those found to be enriched with *cis*-regulatory sequences in humans compared with chimpanzees, indicating that this type of regulation may be particularly effective within the ACC (Haygood et al. 2007). Differential expression of molecules supporting oxidative metabolism and behavior and perception in CN may underlie the connectivity and integration of sensory information involved in language production in humans (Enard 2011).

Importantly, this work identifies categories of biological function whose constituent molecules may be the targets of species-specific posttranscriptional regulation. Our results indicate that the relationship between transcript and protein abundance differs with functional category. Not only did we find a broad variation of slopes in our regression analyses of protein expression on gene transcript expression, but also genes and proteins supporting several functional categories, including those that support translation, protein modifications, biosynthesis of macromolecules, and cellular adhesion, have a stronger correlation than the typical transcript parent/protein product pair. Because the expression of genes within these categories of biological function suggests regulation by a coordinated mechanism, transcript–protein pairs within these categories may offer potential places to explore posttranslational regulation.

There are at least three directions where our knowledge of comparative molecular biology of the human brain is lacking as it relates to other primates. First, it remains unknown the extent to which the relationship between transcript expression and protein expression differs across disparate regions of the brain. Although fewer differences in transcript abundance are seen across specific regions of the cerebral cortex than between the cortex, CN, and cerebellum (Khaitovich et al. 2004; Oldham et al. 2006), the lack of correlated expression between transcripts and proteins draws into question whether protein expression alone would follow a similar trend. Although layer-specific analyses of gene expression patterns in the primate cerebral cortex are now possible (Bernard et al. 2012; Hawrylycz et al. 2012), relatively little is known about the spatial specificity of protein expression. Such analyses of regional transcriptomic and proteomic expression patterns in the brains of humans (and nonhuman primates) are particularly important in light of new evidence that individual neurons carry different genomes comprised transcript sequence repeats or deletions (McConnell et al. 2013) and that sampling large regions of cortex may dilute molecular signals unique to cortical layers or individual cells. Second, because regulation of transcription expression is dynamic over the course of the lifetime (Lu et al. 2004; Somel et al. 2009, 2010; Wei et al. 2015), a better understanding of how transcript and protein expression vary throughout the lifetime, to support neurodevelopmental processes (Liu et al. 2012), should be appreciated. Third, although gene transcripts have been assessed using coexpression networks (Oldham et al. 2008; Winden et al. 2009), the lack of coordination between gene transcripts and protein expression indicates that similar work could be a fruitful contribution to our understanding of the networks of interacting proteins underlying phenotypes.

## Conclusion

In summary, our work provides novel insights into gene and protein expression in the brains of humans and chimpanzees. The low correspondence between transcript and protein expression levels means that different biological signals are reflected in the analysis of one molecule compared with the other. Although the relationship between gene and protein expression is weak overall, we found different, and sometimes stronger, relationships when examining genes and proteins that support specific biological functions. Gene transcript and protein pairs that display different patterns of expression compared with the rest of the transcriptome and proteome may assist in directing future studies in finding regulatory elements that are important in determining the phenotype of the human brain.

## Supplementary Material

Supplementary material, text, data set S1, figures S1–S4, and tables S1–S5 are available at *Genome Biology and Evolution* online (http://www.gbe.oxfordjournals.org/).

Supplementary Data
