# Supplementary material for: Evolutionary Divergence of Gene and Protein Expression in the Brains of Humans and Chimpanzees
Source: Genome Biol Evol. 2015 Jul 10;7(8):2276–88. doi: 10.1093/gbe/evv132 (PMC4558850; doi:10.1093/gbe/evv132)
Supplement: Supplementary Data [file supp_7_8_2276__index.html]

Evolutionary Divergence of Gene and Protein Expression in the Brains of Humans and Chimpanzees — Supplementary Data 

# Evolutionary Divergence of Gene and Protein Expression in the Brains of Humans and Chimpanzees

## Supplementary Data

files

- Supplementary Data - xlsx file
- Supplementary Data - pdf file
- Supplementary Data - xlsx file
- Supplementary Data - xlsx file
- Supplementary Data - xlsx file
