# Supplementary material for: Evolutionary Divergence of Gene and Protein Expression in the Brains of Humans and Chimpanzees
Source: Genome Biol Evol. 2015 Jul 10;7(8):2276–88. doi: 10.1093/gbe/evv132 (PMC4558850; doi:10.1093/gbe/evv132)
Supplement: Supplementary Data [file supp_evv132_Bauernfeind-SupplementaryMaterial-clean.pdf]

Sample preparation for proteomic analysis

Tissue was washed with 70% cold ethanol (with a vortex run between each wash), and then 8 M urea in 50 mM ammonium bicarbonate was added (100 µl per 10 mg of tissue wet weight). Solubilization was accomplished by burst sonication (5 x 3 s at 30% power) with cooling between bursts. The samples were spun at 11,700g for 10 min to remove insoluble material and supernatants were subjected to methanol precipitation (4x the initial urea volume) with incubation for 10 min at 30°C and cooling on ice for 20 min. The samples were then spun and pellets washed with 1 ml methanol. The pellet was resuspended in 500 ml methanol through sonication (3 x 5 s) and protein was pelleted by centrifugation at 5000g for 5 min. The final protein pellet was then resuspended in 0.25% Rapigest (Waters) in 50 mM ammonium bicarbonate (pH 8), vortexed, and incubated at 60°C for 5 min and then subjected to a final centrifugation step to remove insoluble material. Protein concentrations were measured using a Bradford assay. From the isolated protein, 50 mg was removed and normalized to 1 µg/µl prior to reduction, alkylation and trypsin digestion following a standardized protocol (http://www.genome.duke.edu/cores/proteomics/sample-preparation/documents/In-solutionDigestionProtocol_012309.doc). Following digestion, Rapigest surfactant was hydrolyzed by the addition of 1% TFA, pH 2.5 for 2 h at 60°C and non-soluble surfactant was removed by centrifugation. All samples were then spiked with ADH1_YEAST digest (Massprep standard, Waters Corporation) as a surrogate standard (50 fmol ADH per µg total brain lysate). A separate "QC (quality control) pool" was generated by removing an equal quantity (5 µg) from each of the 12 samples.

Quantitative analysis of human and chimpanzee brain proteomes

Quantitative LC/MS/MS was performed on 1 μg of protein digest per sample, using a nanoAcquity UPLC system (Waters Corporation) coupled to a Synapt G2 HDMS high resolution accurate mass tandem mass spectrometer (Waters Corporation) via a nanoelectrospray ionization source. The sample was first trapped on a Symmetry C18 300 mm × 180 mm trapping column (5 μl/min at 99.9/0.1 v/v water/acetonitrile), after which the analytical separation was performed using a 1.7 μm Acquity BEH130 C18 75 mm × 250 mm column (Waters Corporation) using a 90-min gradient of 5 to 40% acetonitrile with 0.1% formic acid at a flow rate of 300 nl/min with a column temperature of 45°C. Data collection on the Synapt G2 mass spectrometer was performed in ion-mobility assisted data-independent acquisition (HDDIA or HDMSE) mode, using 0.6 s alternating cycle time between low (6 V) and high (27-50 V) collision energy. Scans performed at low collision energy measure peptide accurate mass and intensity (abundance), while scans at elevated collision energy allow for qualitative identification of the resulting peptide fragments via database searching. The total analysis cycle time for each sample injection was approximately 2 h.

All samples were run back-to-back following a randomization order of injection. The QC pool sample was run three times throughout the block (including the first and last injections) to obtain QC reproducibility metrics (for a total of 15 quantitative analyses). Following the 15 analyses, data was imported into Rosetta Elucidator v3.3 (Rosetta Biosoftware), and all LC-MS files were aligned based on the accurate mass and retention time of detected ions ("features") using PeakTeller algorithm (Elucidator). The relative peptide abundance was calculated based on area-under-the-curve of aligned features across all runs. The dataset had 474,762 quantified features and high collision energy (peptide fragment) data was collected in 422,513 spectra for

sequencing by database searching, respectively. This MS/MS data was searched against a custom SwissProt/Trembl database, which contained both human (*Homo sapiens*; 26,107 forward entries) and chimpanzee (*Pan troglodytes*; 32,369 forward entries) proteins. Because chimpanzee protein sequences are less well known than the human proteome (despite the larger number of sequences in the online databases), combining the human and chimpanzee databases allowed us to have the most current and detailed database for protein identification. This database was then appended with a decoy reverse-sequence of each forward entry for false positive rate determination. After individual peptide scoring using PeptideProphet algorithm (Elucidator), the data was annotated at a <1% peptide false discovery rate. This analysis yielded identifications for 8,850 peptides and 1,348 proteins across samples, including 858 proteins with two or more peptides quantified. For quantitative processing, the data was first curated to contain only high quality peptides with appropriate chromatographic peak shape and the dataset was intensity scaled to the robust mean across all samples analyzed; the final quantitative dataset was based on 8,775 peptides and contained 1,337 proteins.

Reproducibility of proteomic analysis

Based on Bradford assays, the total quantity of protein ranged from 429 to 514 mg with no apparent outliers based on protein concentrations ranges (Supplementary Figure 1A). To screen for potential outliers, each individual sample was plotted on a principal component analysis (PCA) plot for the top three principal components (Supplementary Figure 1B) based on z-scored transformed (measurement of the significance of change) protein intensity. The PCA did not indicate any analytical outliers in relation to the other samples and all intra-treatment group replicates had similar PC1 (most significant factor) separations. Importantly, we noted that the QC pools (yellow dots; Supplementary Figure 1B) grouped tightly and were located in the

middle of the principal components space, as would be expected since the QC pools were generated from a mixture of all samples. The tight grouping of QC pools reflects a high analytical reproducibility. The reproducibility of the protein expression measurements (technical reproducibility) was assessed by measuring the variation in the protein intensities across the QC pool samples run periodically throughout the run block. In this QC pool sample, the mean CV of protein intensity was 7.4% with a median of 5.2% across the 1337 proteins quantified. As expected, the variation in each of the four treatment groups were slightly higher due to biological and preparation variation (mean variation was 12.7% in human ACC, 20.1% in human CN, 16.0% in chimpanzee ACC, and 18.0% in chimpanzee CN). In both species, there appeared to be slightly more variation among CN samples compared to ACC samples.

As an additional way of assessing variability and demonstrating reproducibility among samples, we found the mean expression level of each peptide per species. Across regions and species, a similar amount of variation exists in peptide expression levels, and as expected, less variation is observed in the QC pool sample (Supplementary Figure 2A). Mean peptide expression levels for both species combined are similar in ACC and CN (Supplementary Figure 2B). Also, the $\log_2$ fold change of peptide expression between species is similar in ACC and CN (Supplementary Figure 2C).

In order to confirm that the protein quantifications were consistent with those of their corresponding peptides, we found $\log_2$ fold change ratios between humans and chimpanzees and between ACC and CN of both peptides and proteins. In each comparison, the correlations between the fold changes of peptide and protein expression displayed moderately high, positive correlations (Pearson correlation of human ACC to chimpanzee ACC Spearman rank rho = 0.56,

$p < 0.0001$; human CN to chimpanzee CN rho = 0.51, $p < 0.0001$; human ACC to human CN rho = 0.57, $p < 0.0001$; chimpanzee ACC to chimpanzee CN rho = 0.52, $p < 0.0001$), displaying consistency between species and regions.

Analysis of the unpaired (complete) dataset

The interindividual variation in molecular expression was assessed by comparing the distribution of the interindividual CVs for the expression of transcripts and proteins. The results of Mann-Whitney tests for differences in central tendency and Kolmogorov-Smirnov tests for differences in the shape of the distributions between transcript and protein expression, regions, and species are listed in Supplementary Table 4 and are presented in Supplementary Figure 3. The distributions of the interindividual variation in transcript expression are very similar between the paired and unpaired datasets, indicating that coding and noncoding transcripts display similar patterns in variation in each brain region and species.

Differential expression of transcripts in the unpaired dataset

We assayed expression from 10,400 genes that were expressed in both the ACC and CN. We found 720 genes to be DE between humans and chimpanzees in the ACC (FDR ≤ 0.05). In contrast, we found far fewer genes as DE in the CN (66 genes; FDR ≤ 0.05). This might be due to large variation in this region within species, possibly due to sex-specific differences. Interestingly, the top two differentially expressed genes in both regions were R-spondin (*RSPO1*; involved in the inhibition of the Wnt/β-Catenin Signaling pathway and involved in cell migration and polarity) and *LIX1*, and both genes are expressed in the brain (Kamata et al., 2004; Moeller et al., 2002). The correlation of differential expression between the ACC and the CN was also

low generally (Spearman rank rho = 0.21, $p < 0.0001$), supporting the idea that these regions display unique specializations of biological functions between humans and chimpanzees.

Gene transcripts supporting 373 categories of biological function in ACC and 140 in CN were found to be DE between humans and chimpanzees (minimum of 10 genes per category, $q \leq 0.05$). Categorical enrichment analyses (Supplementary Figure 4; Supplementary Table 5) show that transcripts involved in neuronal communication, including cellular communication and signal transduction, and structural development (and expressed here in adult tissue), including anatomical structure development and developmental processes are among the most enriched genes displaying differential expression between species in the ACC. For the CN, transcripts involved in intracellular signaling and transport, including multicellular organismal processes, and cellular adhesion, including the categories of cell adhesion and biological adhesion, are among the most enriched genes displaying interspecific differential expression. Although there are many functional categories of genes that show differential expression in both ACC and CN, including those involved in structural development, intracellular signaling, and cell adhesion, several categories are also unique to the region. Specifically, transcripts involved in neuronal communication and cellular regulatory processes are uniquely DE between species in the ACC and may underlie synaptic transmission (Uddin et al., 2004). In contrast, our analysis of interspecific differential expression finds that genes involved in cognition and perception differentiate human and chimpanzee CN. This result is not surprising due to the numerous connections of primary somatosensory cortex to the CN but difficult to interpret due to the unique somatotopic mapping of the region, which is not tracked in our samples (Flaherty & Graybiel, 1993; Geradin 2003).

Differential expression of proteins in the unpaired dataset

We found 67 of the 715 homologous proteins to be differentially expressed between humans and chimpanzees in the ACC (FDR ≤ 0.05). Like the gene expression data, fewer proteins were DE in the CN (58 proteins; FDR ≤ 0.05) compared to ACC. Also seen in the transcript data, the top two differentially expressed proteins were shared by both regions: serum albumin (ALB) and myosin light chain 6B (MYL6B), a blood plasma protein and an ATPase cellular motor protein, respectively. The correlation of differential expression in proteins was more similar between the ACC and the CN (Spearman rank rho = 0.43, $p < 0.0001$) than compared to the genomic data, a result that is not surprising considering the smaller range of variability across protein expression.

Categorical enrichment analyses of proteins in the unpaired dataset (Supplementary Figure 4; Supplementary Table 5) revealed similar findings to that performed on the subset of data from the paired dataset. Fewer biological functions met our threshold criteria (51 in ACC and 26 in CN) for differential expression in humans and chimpanzees from proteins, even though we used more lenient thresholds (minimum of 3 genes per category, $q \leq 0.05$), by nature of having far fewer proteins in our dataset compared to transcripts. Specifically, proteins involved in the oxidative metabolism, including oxidative reduction and metabolic processes, and anaerobic metabolism and biosynthesis, including carbohydrate catabolic processes and cellular carbohydrate catabolic processes, are among the most enriched proteins displaying differential expression between species in the ACC. For the CN, proteins involved in intracellular signaling and transport, including multicellular organismal processes, and cellular adhesion, including the categories of cell adhesion and biological adhesion, are among the most enriched proteins displaying interspecific differential expression. While categories of biological function that

support biosynthesis, perception, and immune response are DE in ACC and CN, proteins involved in anaerobic metabolism are uniquely DE between species in the ACC and may assist in biomolecular turnover that may be unique to one species (Bauernfeind et al., 2014).

References

Bauernfeind AL, et al. 2014. Aerobic glycolysis in the primate brain: reconsidering the implications for growth and maintenance. Brain Struct Funct. 219:1148-1167.

Flaherty AW, Grabiel AM. 1993. Two input systems for body representations in the primate striatal matrix: experimental evidence in the squirrel monkey. J Neurosci. 13:1120-1137.

Geradin E. 2003. Foot, hand, face and eye representation in the human striatum. Cereb Cortex 13:162-169.

Kamata T, et al. 2004. *R-spondin*, a novel gene with thrombospondin type 1 domain, was expressed in the dorsal neural tube and affected in *Wnt*s mutants. Biochim Biophys Acta 1676:51-62.

Moeller C, Yaylaoglu MB, Alvarez-Bolado G, Thaller C, Eichele G. 2002. Murine *Lix1*, a novel marker for substantia nigra, cortical layer 5, and hindbrain structures. Gene Expr Patterns 1:199-203.

Uddin M, et al. 2004. Sister grouping of chimpanzees and humans as revealed by genome-wide phylogenetic analysis of brain gene expression profiles. Proc Natl Acad Sci USA 101:2957-2962.

Supplementary Dataset Legend

Supplementary Dataset 1 – Raw peptide and protein quantifications and individual measures of gene and protein expression for human and chimpanzee ACC and CN. The species means, SDs, and interindividual CVs are included.

Supplementary Table Legends

Supplementary Table 1 – Demographic data for the individuals used in this study.

Species	Individual	Age	Sex	Region
Homo sapiens	1	50.4	F	CN
Homo sapiens	2	34.3	F	ACC, CN
Homo sapiens	3	48.4	F	ACC, CN
Homo sapiens	4	51.2	M	ACC
Pan troglodytes	1	28.9	M	ACC, CN
Pan troglodytes	2	22.5	F	ACC, CN
Pan troglodytes	3	34.5	M	ACC, CN

Supplementary Table 2 – The GO categories of biological function that are supported by the transcripts that are not measured at the protein level, and vice versa. (Table is uploaded as a separate file.)

Supplementary Table 3 – The results of OLS regressions of gene and protein pairs ascribed to a GO category of biological function. (Table is uploaded as a separate file.)

Supplementary Table 4 – The results of Mann-Whitney and Kolmogorov-Smirnov tests of interindividual CVs between gene and protein expression, regions of the brain, and species in the unpaired dataset.

		**Mann-Whitney**		**Kolmogorov-Smirnov**	
**Comparison**		**U**	**p-value**	**D**	**p-value**
Genes vs. proteins	Human ACC	8.2 x 106	< 0.001	0.71	< 0.001
	Human CN	7.2 x 106	< 0.001	0.57	< 0.001
	Chimpanzee ACC	6.7 x 106	< 0.001	0.38	< 0.001
	Chimpanzee CN	7.8 x 106	< 0.001	0.71	< 0.001
ACC vs. CN	Human genes	7.1 x 107	< 0.001	0.05	< 0.001
	Chimpanzee genes	1.2 x 108	< 0.001	0.45	< 0.001
	Human proteins	3.3 x 105	< 0.001	0.24	< 0.001
	Chimpanzee proteins	2.9 x 105	< 0.001	0.12	< 0.001
Humans vs. chimpanzees	Genes in ACC	1.1 x 108	< 0.001	0.31	< 0.001
	Genes in CN	5.2 x 107	< 0.001	0.19	< 0.001
	Proteins in ACC	2.2 x 105	< 0.001	0.12	< 0.001
	Proteins in CN	2.6 x 105	0.51	0.03	0.89

Supplementary Table 5 – DE gene transcripts and protein products between humans and chimpanzees in the GO category of biological function in ACC and CN for the unpaired dataset. The threshold q-values and minimum numbers of genes or proteins per category are listed in each subheading. (Table is uploaded as a separate file.)

Supplementary Figure Legends

Supplementary Figure 1 – Results of tests for protein quality. (A) Bradford assay results following tissue homogenization with burst sonification in the presence of urea. (B) A 3-dimensional principal components analysis of z-score corrected protein expression values from 3 human ACC, 3 human CN, 3 chimpanzee ACC, 3 chimpanzee CN, and 3 QC pools. The QC pools contained equal quantities of each of the 6 human and 6 chimpanzee samples.

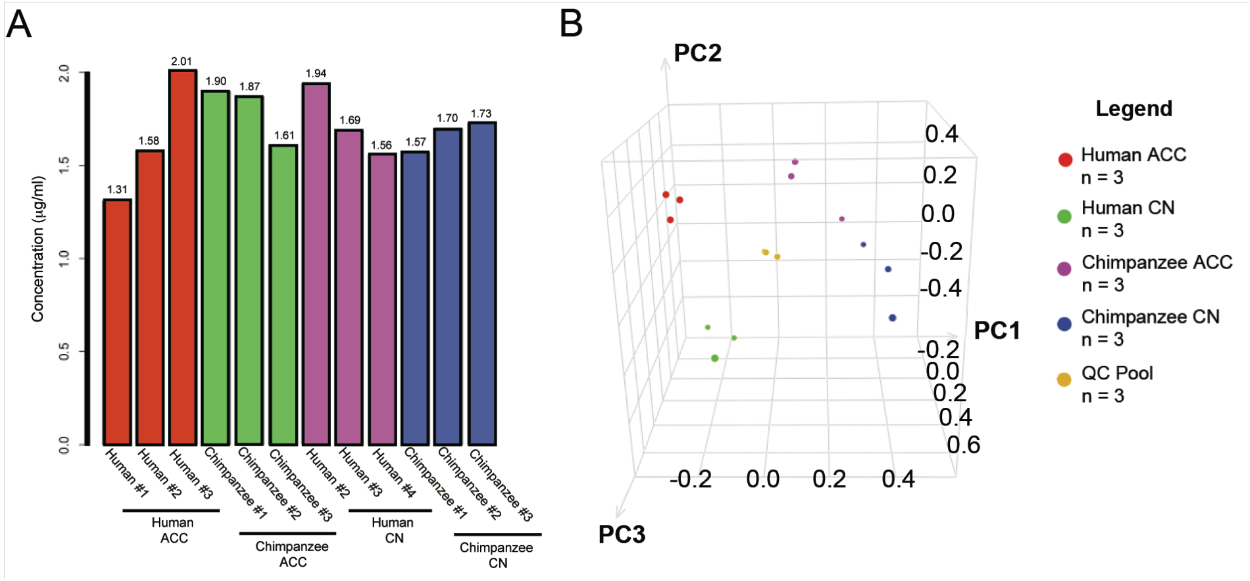

Supplementary Figure 2 – Tests variability of peptide expression quality. (A) Percent standard deviation for each peptide across species, regions, and the QC pool. (B) Percent standard deviation for average peptide expression in humans and chimpanzees combined across ACC and CN. (C) Log_2 fold change ratio of peptide expression between humans and chimpanzees.

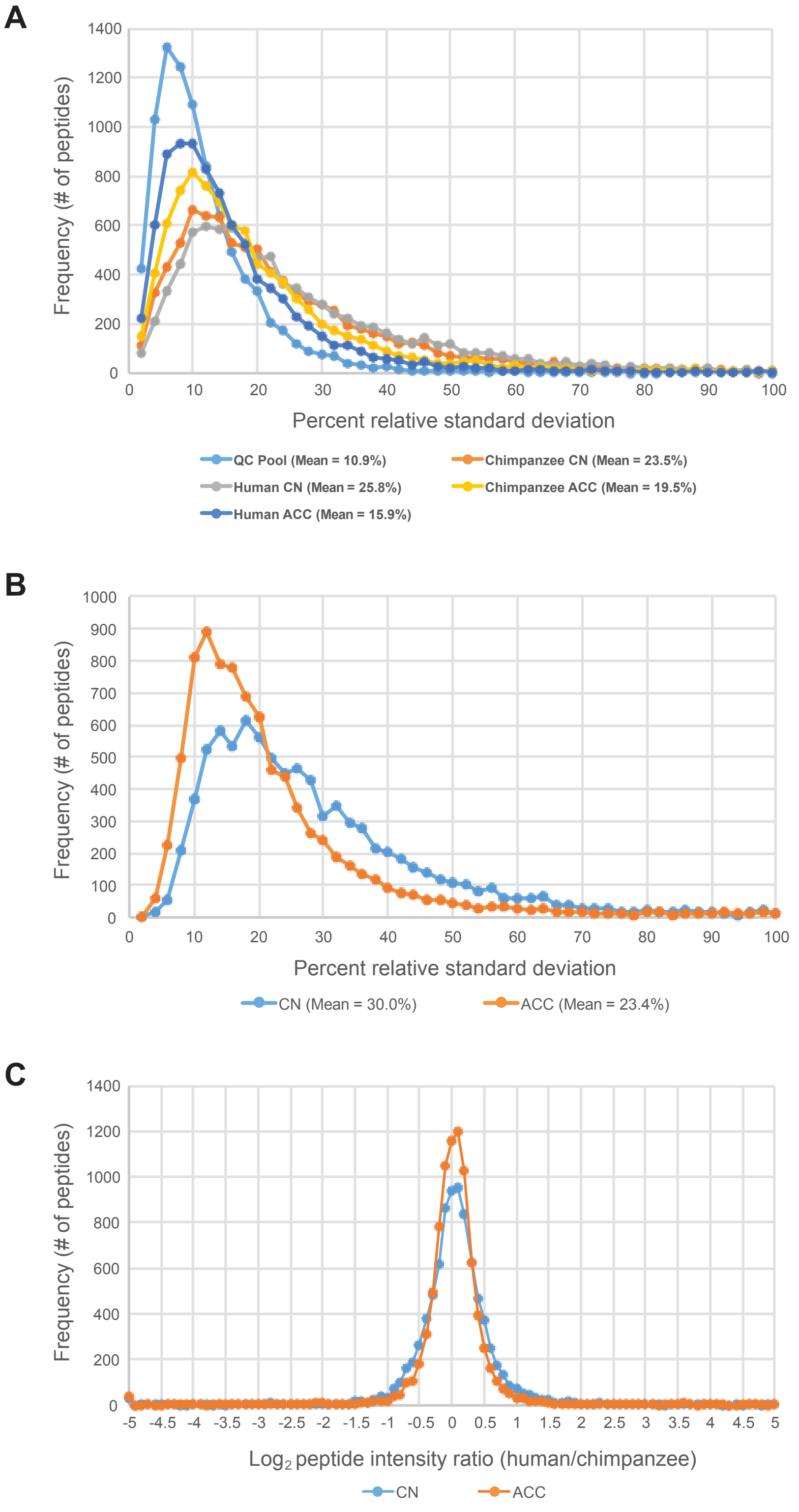
A
Frequency (# of peptides)
Percent relative standard deviation
QC Pool (Mean = 10.9%)
Chimpanzee CN (Mean = 23.5%)
Human CN (Mean = 25.8%)
Chimpanzee ACC (Mean = 19.5%)
Human ACC (Mean = 15.9%)
B
Frequency (# of peptides)
Percent relative standard deviation
CN (Mean = 30.0%)
ACC (Mean = 23.4%)
C
Frequency (# of peptides)
Log_2 peptide intensity ratio (human/chimpanzee)
CN
ACC

Supplementary Figure 3 – Frequency bar graphs of interindividual CVs using the unpaired datasets. The distributions of the coefficients of variation for gene (blue) and protein (red) expression is shown for ACC and CN in humans and chimpanzees. The overlap between these two distributions appears as a darker (purplish) color. The results of the Mann-Whitney tests comparing the measures of central tendency of gene transcripts and proteins are provided.

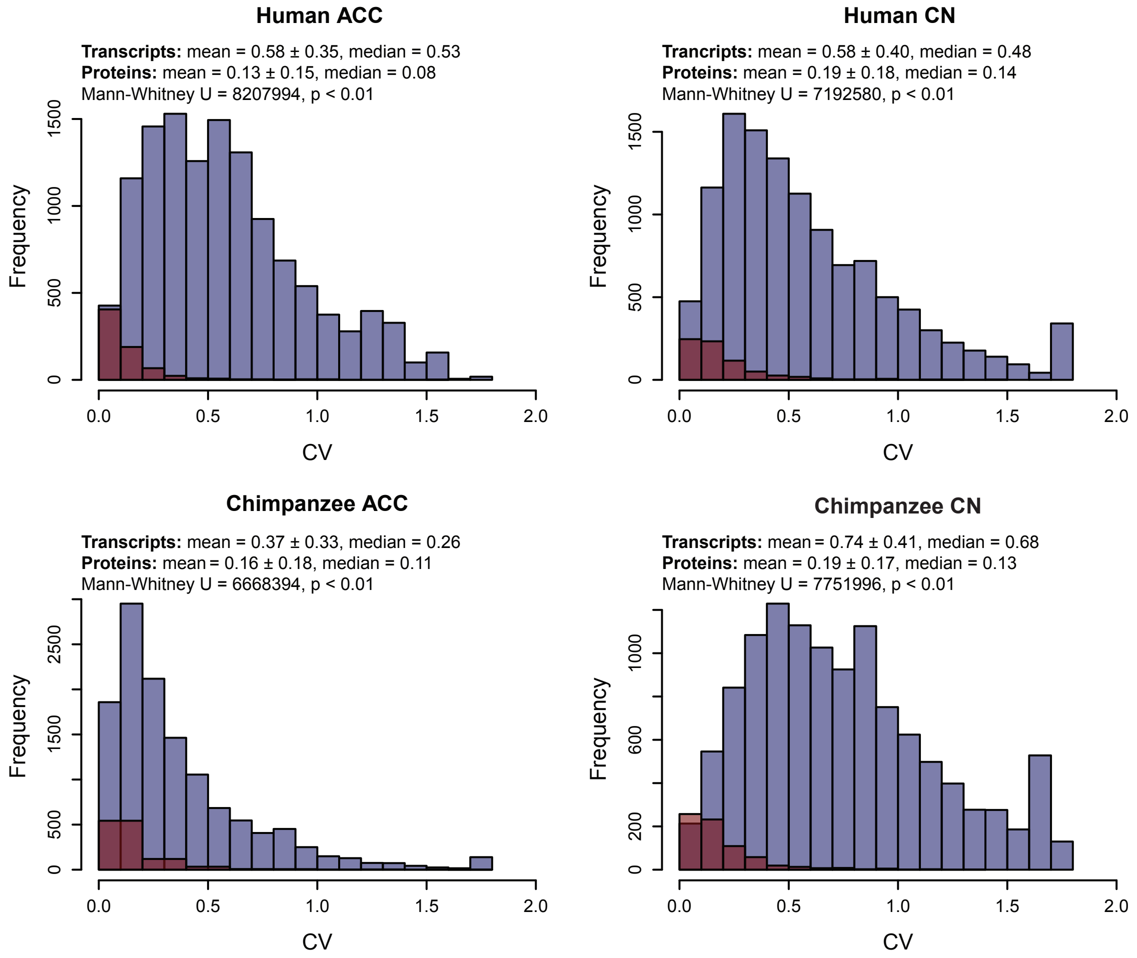

Supplementary Figure 4 – DE gene transcripts and protein products between humans and chimpanzees in GO categories of biological function for the unpaired dataset. The DE categories of transcripts (upper row) are depicted by blue circles for the ACC (upper left) and CN (upper right). The DE categories of proteins (lower row) are depicted by red circles for the ACC (lower left) and CN (lower right). The circles represent categories of biological function, which contain gene transcripts that are DE between the two species. The size of the circle represents the number of genes with a q-value below the maximum threshold (the gray circle in the bottom left corners provide a guide). The darkness of the circle represents the level of significance (as indicated by the scales, which are the same for both ACC and CN). Aside from the degree of overlap of functional categories, the arrangement of the circles has no meaning. The minimum thresholds are different for genes (minimum of 10 genes per category; $q \leq 0.01$) and proteins (in ACC, minimum of 5 proteins per category, $q \leq 0.01$; in CN minimum of 3 proteins per category, $q \leq 0.05$).

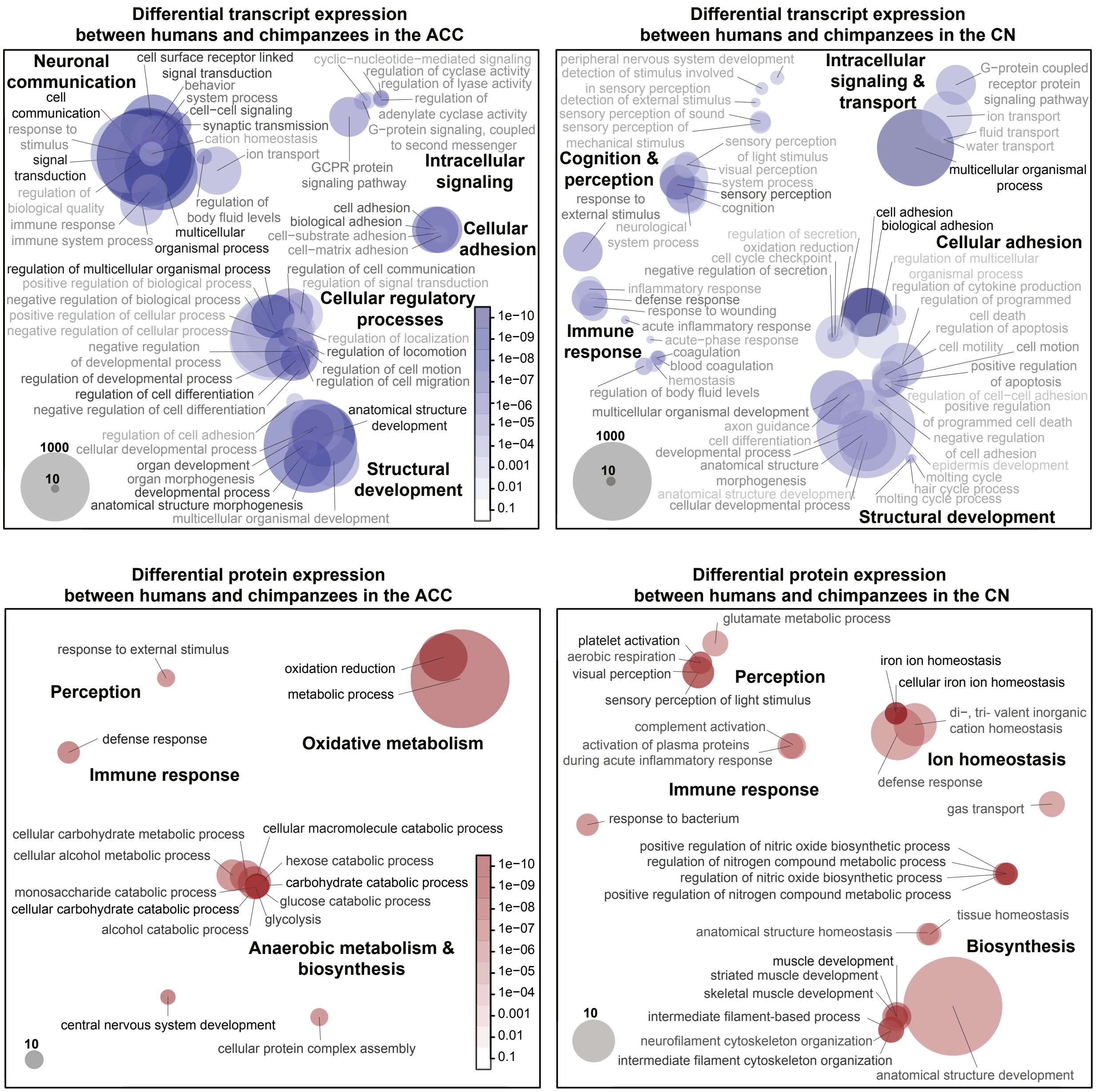
Differential transcript expression
between humans and chimpanzees in the ACC
Neuronal communication
cell surface receptor linked signal transduction
behavior
system process
cell–cell signaling
synaptic transmission
cation homeostasis
ion transport
cell communication
response to stimulus
signal transduction
regulation of biological quality
immune response
immune system process
regulation of body fluid levels
multicellular organismal process
cyclic−nucleotide−mediated signaling
regulation of cyclase activity
regulation of lyase activity
regulation of adenylate cyclase activity
G−protein signaling, coupled to second messenger
GCPR protein signaling pathway
Intracellular signaling
cell adhesion
biological adhesion
cell−substrate adhesion
cell−matrix adhesion
Cellular adhesion
regulation of multicellular organismal process
positive regulation of biological process
negative regulation of biological process
positive regulation of cellular process
negative regulation of cellular process
negative regulation of developmental process
regulation of developmental process
regulation of cell differentiation
negative regulation of cell differentiation
regulation of cell communication
regulation of signal transduction
Cellular regulatory processes
regulation of localization
regulation of locomotion
regulation of cell motion
regulation of cell migration
anatomical structure development
Structural development
regulation of cell adhesion
cellular developmental process
organ development
organ morphogenesis
developmental process
anatomical structure morphogenesis
multicellular organismal development
1000
10
1e−10
1e−09
1e−08
1e−07
1e−06
1e−05
1e−04
0.001
0.01
0.1
Differential transcript expression
between humans and chimpanzees in the CN
peripheral nervous system development
detection of stimulus involved in sensory perception
detection of external stimulus
sensory perception of sound
sensory perception of mechanical stimulus
sensory perception of light stimulus
visual perception
system process
sensory perception
cognition
Cognition & perception
response to external stimulus
neurological system process
Intracellular signaling & transport
G−protein coupled receptor protein signaling pathway
ion transport
fluid transport
water transport
multicellular organismal process
regulation of secretion
oxidation reduction
cell cycle checkpoint
negative regulation of secretion
inflammatory response
defense response
response to wounding
acute inflammatory response
acute−phase response
coagulation
blood coagulation
hemostasis
regulation of body fluid levels
Immune response
cell adhesion
biological adhesion
Cellular adhesion
regulation of multicellular organismal process
regulation of cytokine production
regulation of programmed cell death
regulation of apoptosis
cell motility
cell motion
positive regulation of apoptosis
regulation of cell−cell adhesion
positive regulation of programmed cell death
negative regulation of cell adhesion
epidermis development
molting cycle
hair cycle process
molting cycle process
multicellular organismal development
axon guidance
cell differentiation
developmental process
anatomical structure morphogenesis
anatomical structure development
cellular developmental process
Structural development
1000
10
Differential protein expression
between humans and chimpanzees in the ACC
response to external stimulus
Perception
defense response
Immune response
oxidation reduction
metabolic process
Oxidative metabolism
cellular carbohydrate metabolic process
cellular alcohol metabolic process
monosaccharide catabolic process
cellular carbohydrate catabolic process
alcohol catabolic process
cellular macromolecule catabolic process
hexose catabolic process
carbohydrate catabolic process
glucose catabolic process
glycolysis
Anaerobic metabolism & biosynthesis
central nervous system development
cellular protein complex assembly
10
1e−10
1e−09
1e−08
1e−07
1e−06
1e−05
1e−04
0.001
0.01
0.1
Differential protein expression
between humans and chimpanzees in the CN
glutamate metabolic process
platelet activation
aerobic respiration
visual perception
sensory perception of light stimulus
Perception
complement activation
activation of plasma proteins during acute inflammatory response
Immune response
response to bacterium
iron ion homeostasis
cellular iron ion homeostasis
di−, tri− valent inorganic cation homeostasis
Ion homeostasis
defense response
gas transport
positive regulation of nitric oxide biosynthetic process
regulation of nitrogen compound metabolic process
regulation of nitric oxide biosynthetic process
positive regulation of nitrogen compound metabolic process
tissue homeostasis
anatomical structure homeostasis
Biosynthesis
muscle development
striated muscle development
skeletal muscle development
intermediate filament-based process
neurofilament cytoskeleton organization
intermediate filament cytoskeleton organization
anatomical structure development
10
